# The contribution of sleep to the neuroendocrine regulation of rhythms in human leukocyte traffic

**DOI:** 10.1007/s00281-021-00904-6

**Published:** 2022-01-18

**Authors:** Tanja Lange, Finn Luebber, Hanna Grasshoff, Luciana Besedovsky

**Affiliations:** 1 Department of Rheumatology and Clinical Immunology, University of Lübeck, Lübeck, Germany; 2 Center of Brain, Behavior and Metabolism (CBBM), University of Lübeck, Lübeck, Germany; 3 Social Neuroscience Lab, University of Lübeck, Lübeck, Germany; 4 Institute of Medical Psychology, LMU Munich, Munich, Germany

**Keywords:** Sleep, Circadian rhythm, Cortisol, Sympathetic nervous system, Immune system, Inflammation

## Abstract

Twenty-four-hour rhythms in immune parameters and functions are robustly observed phenomena in biomedicine. Here, we summarize the important role of sleep and associated parameters on the neuroendocrine regulation of rhythmic immune cell traffic to different compartments, with a focus on human leukocyte subsets. Blood counts of “stress leukocytes” such as neutrophils, natural killer cells, and highly differentiated cytotoxic T cells present a rhythm with a daytime peak. It is mediated by morning increases in epinephrine, leading to a mobilization of these cells out of the marginal pool into the circulation following a fast, beta2-adrenoceptor-dependent inhibition of adhesive integrin signaling. In contrast, other subsets such as eosinophils and less differentiated T cells are redirected out of the circulation during daytime. This is mediated by stimulation of the glucocorticoid receptor following morning increases in cortisol, which promotes CXCR4-driven leukocyte traffic, presumably to the bone marrow. Hence, these cells show highest numbers in blood at night when cortisol levels are lowest. Sleep adds to these rhythms by actively suppressing epinephrine and cortisol levels. In addition, sleep increases levels of immunosupportive mediators, such as aldosterone and growth hormone, which are assumed to promote T-cell homing to lymph nodes, thus facilitating the initiation of adaptive immune responses during sleep. Taken together, sleep–wake behavior with its unique neuroendocrine changes regulates human leukocyte traffic with overall immunosupportive effects during nocturnal sleep. In contrast, integrin de-activation and redistribution of certain leukocytes to the bone marrow during daytime activity presumably serves immune regulation and homeostasis.

## Robust rhythms in human blood leukocyte counts

**Fig. 1:**
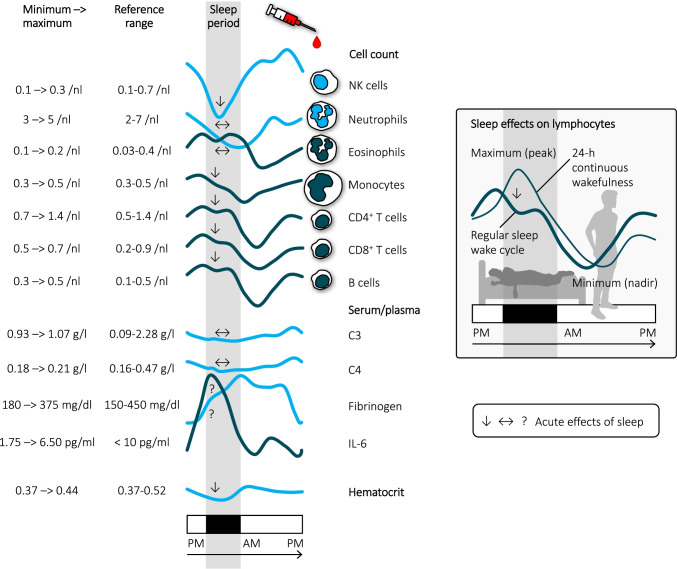
**| Rhythms in human leukocyte subsets.** Shown are schematic 24-h rhythms of white blood cell differential counts [1–3], C3/C4 [5], fibrinogen, IL-6 [4], and hematocrit [1] with tabular listing of minimum/maximum levels and lower/upper normal range. Analyses were performed during a regular sleep–wake cycle; the sleep period is shown in gray, starting from 10:30 to 11 PM and ending 6:30 to 8:30 AM (depending on the study) [1–5]. The rhythm of lymphocyte counts during continuous wakefulness in comparison to a regular sleep–wake cycle is additionally depicted in the insert [1]. Arrows (or question marks for unclear effects) represent the acute effects of sleep (emerging within the sleep period compared to continuous wakefulness), modulating the rhythms. Note that neutrophils were not acutely affected by sleep in 24-h analyses [1] but were affected in other studies, in particular with periods of prolonged sleep restriction [15]. Parameters peaking during **nocturnal** sleep and **daytime** activity are shown in dark and light blue, respectively

In laboratory medicine, inflammation due to infections, allergy, cancer, or autoimmunity is routinely assessed by a differential white blood cell count, by erythrocyte sedimentation rate or by C-reactive protein (CRP) levels in peripheral blood. More sophisticated blood measurements include flow-cytometric analyses (e.g., of lymphocyte subsets) or the quantification of soluble immune mediators such as procalcitonin (PCT), immunoglobulins (e.g., IgG, IgA), complement factors (e.g., C3, C4), fibrinogen, cytokines (e.g., interleukin-6, IL-6), or their shedded receptors (e.g., soluble IL-2 receptor, sIL-2R). For several of these parameters, **24-h rhythms** have been documented in healthy individuals. Though some of these rhythms are of high amplitude, nadirs and peaks stay within the normal range of reference values (Fig. 1) [e.g., 1–5]. Overall, 24-h rhythms are more robust for circulating leukocyte counts than for soluble mediators. Temporal changes in cell counts mainly reflect acute changes in leukocyte traffic. Rhythms in soluble immune parameters may be more difficult to detect than in leukocytes, because the half-life of the soluble factors is too long (e.g., CRP/PCT ~ 20 h, IgG ~ 25 days) or because their systemic levels are confounded by the procedure of repeated blood sampling in experimental settings (e.g., local release of IL-6 at the site of an intravenous line) [6]. In general, any cellular or soluble parameter in peripheral blood could also change due to plasma shifts into or out of the circulation. In healthy individuals, the hematocrit, which is an indicator of plasma shifts, shows a significant 24-h rhythm, that—although of smaller amplitude than those of immunological parameters—might influence immune rhythms and should be considered in analyses [1].

Given the robust 24-h rhythms in blood counts of leukocyte subsets in humans, our review will focus on the temporal regulation of human immune-cell traffic. We will describe opposing rhythms in different leukocyte subsets and outline that these are driven mainly by the hypothalamus–pituitary–adrenal (HPA)-axis and the sympathetic nervous system (SNS). The mediators of these stress systems—the hormone cortisol and the catecholamines epinephrine and norepinephrine—act on immune cells, which in turn change their expression or function of **pro-migratory molecules**. Notably, the release of cortisol and catecholamines depends on the central pacemaker in hypothalamic suprachiasmatic nuclei (SCN) but is also influenced by environmental, physical, and psychosocial stressors and by behavioral changes. Sleep is an important example of such a behavioral influence: compared to nocturnal wakefulness, sleep further suppresses cortisol and catecholamine levels and thus actively contributes to the rhythmic regulation of leukocyte numbers. The following chapters will therefore focus on this contribution of sleep and describe the underlying hormonal and molecular mediators. Two further chapters briefly summarize the link between rhythmic leukocyte traffic and temporal changes in immune-cell functions and describe further factors influencing immune-cell rhythms. We will then discuss similar as well as discrepant findings in animals, before we close our review with clinical implications and considerations for future experiments. Methodological pitfalls are summarized in a separate box, and some terms (highlighted bold in the text) are further explained in a glossary.

Of note, most of the experimental data in this field was obtained from healthy, young, and predominantly male adults. However, the **circadian system**, sleep, stress mediators, and immune parameters differ between males and females and change across the lifespan. It is beyond the scope of this review to discuss in detail the effects of sex and age in this context. Therefore, the interested reader is referred to other publications discussing these effects in more depth [e.g., 7] (see also chapters “Other time dimensions and influences relevant for immune-cell rhythms” and “Conclusions, clinical implications, and outlook”).

## Opposing rhythms in human T-cell subsets have been known for decades

Already in the 1980s, Abo et al. reported opposing rhythms in numbers of T-cell subsets in human blood with “Tμ cells” peaking during the night and “Tγ cellsˮ peaking during the day [8]. Tμ cells were identified by IgM-induced rosette forming of sheep red blood cells and made up about 50% of lymphocytes. On the other hand, the smaller population of Tγ cells was identified by IgG-induced rosette forming [9]. Compared to Tμ-cells, Tγ-cells did not recirculate through lymph nodes and thoracic duct, were bigger, and showed a shorter half-life and a higher degree of differentiation. They were later reported to consist of only one quarter of “true T cellsˮ and otherwise of natural killer (NK) cells [10]. Several decades later, opposing rhythms were confirmed in human lymphocyte subsets, which were identified by multiparametric flow cytometry with staining for surface markers (cluster of differentiation, CD) that are necessary for the recirculation between blood and lymph nodes (e.g., CD62L, CCR7) or characterize cell differentiation and cytotoxicity (e.g., CD45RA, CD16, CD56) [11–13]. Findings indicated that Tμ cells are identical to naive and central memory CD4^+^ and CD8^+^ T cells, which are T cells at early stages of differentiation. In contrast, Tγ cells encompass highly differentiated cytotoxic effector cells such as CD8^+^ effector memory T cells re-expressing CD45RA (CD8^+^ Temra), CD4^−^CD8^−^ (double negative) T cells, and NK-like T cells [e.g., 10, 11, 13, 14]. In the following, we will rename Tμ and Tγ cells according to their stages of differentiation with the terms Tearly (naive and central memory T cells) and Tlate (CD8^+^ Temra, double negative, and NK-like T cells), respectively.

## Stress mediators regulate traffic of Tearly and Tlate and of other leukocyte subsets

The HPA-axis and the SNS are the two major stress systems that prepare the organism to fight, flight, or freeze upon an environmental challenge, mainly by mobilizing and re-allocating energy to demanding tissues. As stressors were evolutionary associated with wounding, it is reasonable to activate the immune system to ward off invading pathogens and to initiate tissue healing. Indeed, pro-inflammatory processes during acute stress have been described and seem to involve mainly innate immune cells, as well as atypical **glucocorticoid receptor** (GR)-signaling or α-**adrenoceptors** (αAdR) [16–19]. On the other hand, there are also acute anti-inflammatory, immuno-regulatory effects of stress mediators on innate and adaptive immune cells involving genomic GR-signaling or βAdR [16, 18, 20]. T cells seem to express only βAdR and no αAdR [21] and therefore mainly respond to the anti-inflammatory effects of catecholamines. In the following we will outline the effects of epinephrine and cortisol on pro-migratory molecules, which impact blood leukocyte numbers within minutes and hours, respectively.

### Epinephrine inhibits integrin activation and mobilizes Tlate within minutes

**Fig. 2:**
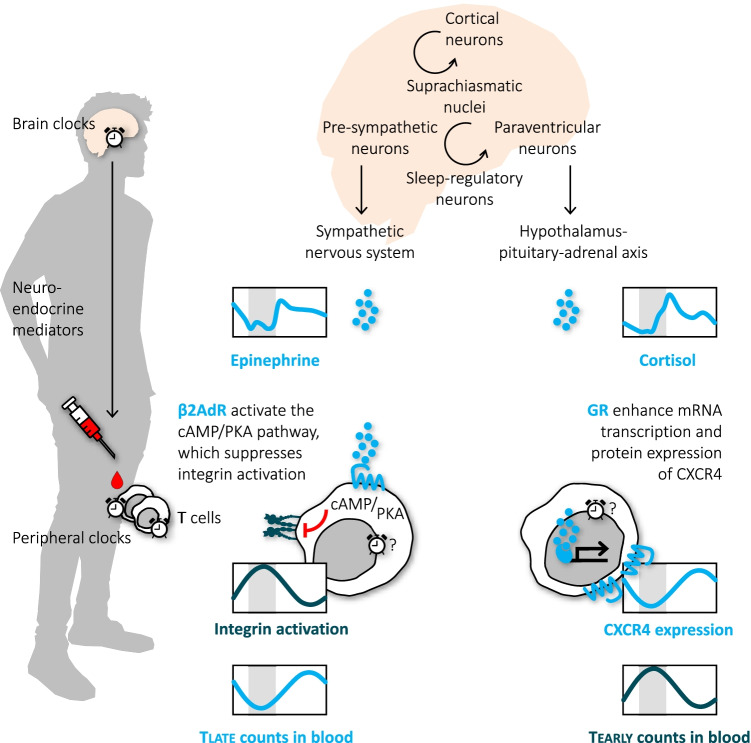
**| Regulation of human T cell traffic by the stress systems.** In the central nervous system, molecular clocks in suprachiasmatic nuclei (SCN) form the master clock, which entrains peripheral clocks via, among others, neuroendocrine mediators, such as epinephrine and cortisol. The SCN interacts with pre-sympathetic and paraventricular neurons and in this way modulates the sympathetic nervous system and the hypothalamus–pituitary–adrenal axis, respectively. Sleep, which interacts with the SCN via cortical and sleep-regulatory neurons, contributes to the rhythmic release of epinephrine and cortisol. These mediators act on T cells in peripheral blood: Epinephrine binds to β_2_-adrenoceptors (AdR), stimulates cyclic adenosine monophosphate (cAMP)/protein kinase A (PKA) signaling, suppresses integrin activation, and mobilizes Tlate into the circulation. Cortisol stimulates glucocorticoid receptors (GR) leading to enhanced CXCR4 mRNA and protein expression and extravasation of Tearly

The most robust immunological phenomenon upon SNS activation is an increase in leukocyte numbers that physicians often recognize in patients as “stress leukocytosisˮ [22]. Stress leukocytosis, also termed “adrenergic leukocytosisˮ has been known since the early 1900s in medicine and human research and describes a rapid increase of circulating leukocyte subset counts in response to a physical or psychosocial stressor. It emerges within minutes, as the fast-acting epinephrine, which is released instantly by the adrenal medulla into the circulation upon sympathetic activation, binds to β_2_AdR on immune cells and mobilizes “stress leukocytesˮ out of the marginal pool into the bloodstream [21]. These stress leukocytes are neutrophils as well as cytotoxic effector cells such as CD16^+^ monocytes, CD16^+^ NK cells, and Tlate. All these subsets increase acutely in healthy individuals also upon experimental epinephrine infusion [14, 23]. During steady-state conditions, stress leukocytes adhere to the endothelium of postcapillary venules of various organs forming the marginal pool. Spontaneous adhesion of CD16^+^ NK cells and Tlate to activated endothelium and de-adhesion by epinephrine was demonstrated in vitro [14]. Several studies in healthy individuals (which phenotyped stress leukocytes and used in vivo and in vitro epinephrine, βAdR agonists or blockers) delineated the pro-migratory molecules that presumably drive this adhesion and de-adhesion processes: It seems that stress leukocytes form the marginal pool when, e.g., fractalkine/CX3CR1 or antigen/T-cell receptor (TRC) signaling activate their integrins (e.g., the β_2_-integrin CD11a/CD18, known as “leukocyte function antigen-1,ˮ LFA-1) and allows binding to corresponding endothelial ligands (e.g., intercellular adhesion molecule-1, ICAM-1). Epinephrine, by stimulating β_2_AdR and the cyclic adenosine monophosphate (cAMP)/protein kinase A (PKA) pathway, immediately suppresses integrin activation and thus leads to leukocyte de-margination [24, 25]. These processes start within seconds [24] and can be monitored by flow cytometry within minutes [25], because de-activation of integrins by cAMP relies on conformational changes of the existing molecule, which do not require protein synthesis [24]. Vice versa, blood numbers of CD8^+^ T cells and NK cells immediately drop when SNS activity is locally suppressed by stellate ganglion block [26]. Together with findings on increases in integrin activation on T cells upon in vitro blockade of the β_2_AdR [25], a tonic inhibition of integrins by endogenous epinephrine can be assumed. Epinephrine-induced integrin de-activation explains the morning rise in stress leukocytes, including CD16^+^ monocytes, CD16^+^ NK cells, and Tlate [11–13, 27], which parallels rhythmic sympathetic activation (Fig. 2). It is unknown whether leukocytes in the marginal pool scan the endothelium for immune challenges in surrounding tissues or whether they simply reside inactively on the endothelium to build up a cellular reservoir that can be mobilized when needed. Whatever the case, since integrin-mediated adhesion is essential for cytotoxic effector cell functions including firm adhesion for subsequent transmigration into tissues and interaction with target cells for the “kiss of deathˮ (i.e., killing of virus-infected cells or tumor cells), the integrin de-activating effects of epinephrine through the β_2_AdR are clearly immunosuppressive and anti-inflammatory actions [25, 28].

### Cortisol upregulates CXCR4 and promotes traffic of Tearly into the bone marrow within hours

Apart from the immediate leukocytosis, acute stress induces a slower, second wave of changes in leukocyte numbers that is driven by cortisol. T-cell numbers decline within 3 h after cortisol administration [13, 29]. In early studies in humans and guinea pigs on stress-induced decreases in T-cell counts, Fauci et al. concluded that glucocorticoids redirect recirculating Tμ/Tearly (but not Tγ/Tlate) out of the blood and secondary lymphatic organs into the bone marrow [30, 31]. This compartment contains high amounts of the chemokine CXCL12/SDF-1, and indeed activation of the GR upregulates expression and signaling of the corresponding chemokine receptor CXCR4 on T cells [32, 33]. GR actions on CXCR4-driven T-cell traffic seem to involve genomic effects enhancing CXCR4 mRNA [34] but also fast non-genomic actions promoting CXCR4 signaling via the Src kinase Lck pathway [33] and suppressing CXCR4 internalization via the cAMP/PKA pathway [35, 36]. The endogenous rise in cortisol in the early morning is therefore followed by enhanced CXCR4 expression on T cells and a concomitant decline in their numbers, which is suppressed if the morning cortisol rise is attenuated [32] (Fig. 2). This decrease is most evident in Tearly, which are easily recruited to the bone marrow as they express highest levels of CXCR4 [13, 32]. Rhythmic CXCR4-driven bone marrow egress and re-entry with higher blood counts in the evening than in the morning were likewise described for hematopoietic stem cells during a regular sleep–wake cycle [37]. Recruitment into the bone marrow might be further boosted by local increases in CXCL12 production by stroma cells during daytime [37]. The functional consequences of T-cell entry into the bone marrow upon GR activation are not known. Long-living Tearly might receive survival signals in the bone marrow niche that could protect against deleterious actions of high levels of glucocorticoids [38]. As their traffic into secondary lymphatic organs at the same time may be impaired [39], it might be a period of suppressed T-cell immunity. However, murine bone marrow T cells can be primed by blood-borne antigens [40] and human bone marrow is a reservoir for memory T cells [38]. Thus, Tearly could rest in the bone marrow during daytime but be awakened by circulating antigens in the case of a major systemic infectious challenge, e.g., following wounding that evolutionary often accompanied the fight-flight-or-freeze response.

### Joint effects of epinephrine and cortisol on traffic of some innate immune cells

While T-cell subsets either respond to epinephrine or to cortisol [13] and accordingly can be dichotomized into epinephrine-sensitive Tlate with a daytime rhythm or cortisol-sensitive Tearly with a nocturnal rhythm [8, 31], other leukocytes, in particular innate immune cells, can react to both stress mediators. Blood numbers of neutrophils increase, whereas eosinophil counts decrease in response to cortisol as well as epinephrine [2, 23]. Epinephrine de-marginates neutrophils [16, 22, 41]. Cortisol additionally contributes to their increase in blood numbers and thus to stress leukocytosis by promoting neutrophil egress out of the bone marrow and preventing their traffic to other peripheral tissues, in particular to inflamed tissues [23, 31, 41]. In eosinophils, like in Tearly, cortisol activating the GR enhances CXCR4 expression [42] and most likely redirects these cells to the bone marrow [43], whereas there is indirect evidence that their CCR3-driven traffic into inflamed tissues is impaired [42]. The mechanisms of epinephrine-induced decreases in eosinophil counts are still obscure, which is remarkable given that the 24-h rhythm in eosinophils is a very robust phenomenon [1–3]. Epinephrine, by activating β_2_AdR and cAMP/PKA, induces further upregulation of CXCR4 as shown in in vitro experiments, potentially by inhibiting its internalization [36]. However, innate immune cells—in contrast to T cells—not only express β_2_AdR but also αAdR, with unclear effects on granulocyte traffic [16, 21]. Overall, further studies assessing AdR types, integrin activation, CXCR4 signaling, adhesion, and migration of eosinophils are necessary to clarify the mechanisms of epinephrine-induced eosinopenia. In intensive care medicine, physicians know that glucocorticoids can increase the sensitivity of AdR towards catecholamines and GR mediated increases in cAMP/PKA signaling might contribute to this phenomenon [35]. Thus, not only additive but also synergistic actions of cortisol and epinephrine on immune cell traffic are conceivable.

## Effects of sleep on rhythms of leukocyte subsets

### Sleep and its regulation

Sleep is a behavioral state characterized by a period of reduced reactivity to stimuli, relative inactivity, loss of consciousness, and (compared to coma) easy reversibility. It can be objectively measured by polysomnography (including electroencephalographic, electrooculographic, and electromyographic recordings), which allows to reliably discriminate between different sleep stages. It typically begins with sleep stage 1 (N1), which is a transitional state between sleep and wakefulness and is followed by light sleep (N2), deep sleep (N3—also called slow-wave sleep, SWS) due to its low-frequency electroencephalographic waves) and finally by rapid eye movement (REM) sleep (R). During the night, 4–5 of such sleep cycles occur and the initial predominance of SWS is replaced by longer periods of REM sleep in the morning hours. According to the two-process model proposed by Borbély first in 1982 [44], sleep regulation occurs by two factors: the homeostatic process S enhances sleep pressure in response to increases in wake duration and primarily promotes SWS following an accumulation of sleep regulatory substances such as adenosine, tumor necrosis factor, and IL-1 during wakefulness. The circadian process C leads to different sleep propensities depending on the time of day. It counteracts process S during daytime as it promotes wakefulness involving alerting neurotransmitters such as norepinephrine, dopamine, serotonin and histamine. In the evening, when process S is high and process C declines, sleep is initiated and shows its typical choreography of N1, N2, N3, and R cycles [45, 46]. The two-process model nicely illustrates that sleep is regulated by the circadian system. On the other hand, sleep can reset cellular clocks in the SCN [47] and the periphery [48], showing the strong bidirectional interaction between these two systems.

### Sleep acutely suppresses levels of epinephrine and cortisol, modulating immune rhythms

**Fig. 3:**
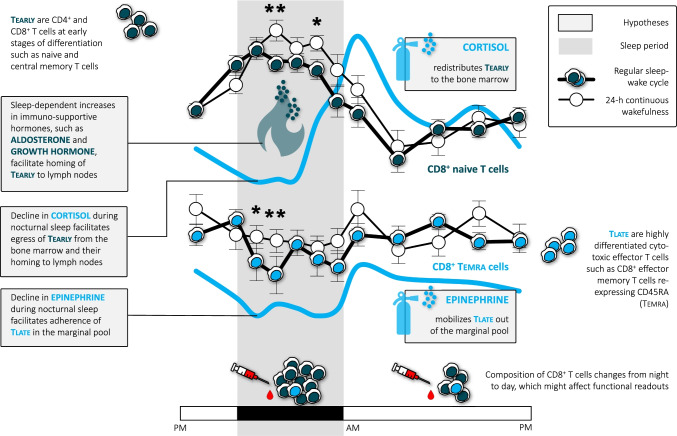
**| Effects of sleep on human T-cell subsets.** CD8^+^ naive T cells, as an example of Tearly, and CD8^+^ effector memory T cells re-expressing CD45RA (Temra), as an example of Tlate, show lower nocturnal counts during a regular sleep–wake cycle (with sleep from 11 PM to 7 AM) compared to continuous wakefulness [27]. Cortisol and epinephrine (here shown schematically for a regular sleep wake cycle [50]) drive the decrease in naive T cells and the increase in Temra in the morning, respectively. Sleep compared to continuous wakefulness suppresses nocturnal levels of cortisol and epinephrine, with the latter explaining the sleep-associated reduction in Temra counts. The unexpected reduction of naive T cell counts during sleep presumably reflects the redistribution of these cells to lymph nodes mediated by reductions in cortisol levels and increases in levels of immunosupportive hormones, such as aldosterone and growth hormone. Overall, this leads to pro-inflammatory effects during nocturnal sleep and immunoregulatory effects during daytime activity, since cells can enter inflamed tissue mainly at times of low cortisol and epinephrine levels. Fourteen healthy young men, within-subjects design, *p < 0.05, ** < 0.01 for comparisons between a regular sleep–wake cycle and 24-h continuous wakefulness. Adapted from [27]

Sleep (versus experimental sleep deprivation) acutely suppresses the SNS and HPA axis (see box for a discussion of methodological issues in this context). During regular sleep, epinephrine levels in healthy individuals show a successive decline from wakefulness to N1, N2, N3, and REM sleep [49]. Parallel assessments of leukocyte numbers in such fine-grained repeated blood draws every 10 min in sleeping individuals are currently missing. However, using 1.5–3-h intervals for blood sampling during a regular sleep–wake cycle, a parallel nocturnal decline in epinephrine [50], CD16^+^ monocytes [12], CD16^+^ NK cells [12], and CD8^+^ Temra [27] followed by a morning rise in these parameters has been documented. These rhythms were markedly attenuated in a condition of continuous wakefulness with participants refraining from locomotor activity or food intake at night [12, 27, 50] (Fig. 3 for CD8^+^ Temra), demonstrating acute regulatory effects of sleep on these parameters. With respect to the HPA axis, the cortisol rhythm is under tight control of the SCN. However, there is a small reduction in cortisol nadir levels during sleep compared to nocturnal wakefulness, which might be missed in infrequent blood sampling protocols [1]. At the same time, there is a steeper increase in cortisol levels in the morning following sleep compared to nocturnal wakefulness. Therefore, sleep enhances the rhythm amplitude of cortisol [5]. The reduction in cortisol levels during sleep should lead to higher bone marrow release and thus to increased blood numbers of Tearly in sleeping compared to awake individuals. Unexpectedly, the opposite holds true: sleep compared to nocturnal wakefulness acutely lowers the peak of circulating Tearly [27] (Fig. 3 for CD8^+^ naive T cells), a robust phenomenon that can also be observed for counts of total, CD4^+^ or CD8^+^ T cells [1, 12, 27]. SWS appears to play a central role in this sleep effect as stimulation of the electrophysiological slow waves can further reduce cortisol levels in sleeping individuals and leads to a parallel drop in blood T-cell numbers [51]. In the next section, we will discuss the assumption that sleep actively lowers blood T-cell counts by promoting the homing of Tearly to lymph nodes.

### Sleep might promote lymph-node homing of Tearly by lowering levels of stress mediators

Tearly recirculate through lymph nodes to receive survival signals and to scan the body for their cognate antigens. We assume that Tearly, after being released from the bone marrow, readily enter the lymph nodes and that this traffic is supported by SWS. Lymph node entry across high endothelial venules (HEV) depends on the selectin CD62L and the chemokine receptor CCR7 (in conjunction with CXCR4), which activates the integrin LFA-1 [52]. All these pro-migratory molecules are expressed on Tearly. GR activation impairs T-cell migration across HEV [39], maybe by decreasing endothelial ICAM-1 expression [53]. Hence, reductions in cortisol and epinephrine levels during sleep—by allowing increases in endothelial ICAM-1 expression and activation of LFA-1 on T cells, respectively—might be a necessary prerequisite for Tearly to enter lymph nodes. In contrast to these effects on ICAM-1, expression of the lymph node homing receptors CD62L and CCR7 was not affected by manipulation of GR on T cells in vivo or in vitro [29]. However, as discussed in the next section, other hormones that are mainly released during SWS may actively support T-cell homing to lymph nodes by promoting CD62L/CCR7 signaling and by maintaining CXCR4 expression levels.

### Further mediators linking sleep and lymph node homing

Sleep not only suppresses levels of stress mediators but also enhances the release of immunosupportive hormones, such as the **mineralocorticoid receptor (MR)** agonist aldosterone and the pituitary hormones growth hormone (GH) and prolactin. All three mediators increase early during sleep, which indicates a stimulatory effect of SWS on their release [5, 12, 54]. In the above-mentioned study, in which slow waves were stimulated, decreases in cortisol levels were accompanied by increases in aldosterone levels and a subsequent decline in T-cell numbers [51]. In vivo and in vitro analyses using MR agonists and antagonists in healthy individuals suggest that MR activation during nocturnal sleep fosters T-cell homing to lymph nodes by increasing CD62L, CCR7, and CXCR4 expression [55, 56]. Like for GR, effects of MR activation were most pronounced for Tearly. In the absence of the enzyme 11β-hydroxysteroid dehydrogenase 2, cortisol can bind to MR with even higher affinity than to GR. Nadir cortisol levels during nocturnal sleep could thus lead to predominant MR activation. However, MR affinity of human peripheral blood mononuclear cells is higher for aldosterone than for cortisol [57]. Therefore, SWS-dependent release of aldosterone activating the MR at a time of minimal cortisol-induced GR activation could be the ideal hormonal constellation for efficient T-cell homing to lymph nodes. In addition to aldosterone, sleep compared to nocturnal wakefulness also increases the release of GH and prolactin [5, 12] and enhances the nocturnal peak of melatonin [5]. All three hormones may also ease T-cell traffic to lymph nodes, although evidence so far is indirect and comes from heterogeneous animal studies [46, 58–60] (Fig. 3).

### Other drivers of immune rhythms are also influenced by sleep

Sleep also prevents behaviors that clearly activate the stress systems. These behavioral changes occur in the morning, when we open our eyes, get out of bed and thus switch from a recumbent to an upright posture, start locomotor activity or even physical exercise, drink, eat, and expose ourselves to noxious agents (e.g., smoking, particulate matter), physical stimuli (e.g., sunlight, ambient temperature, noise) or psychosocial stimuli (e.g., interactions with other people). While positive social interactions may have stress-reducing effects, they can also lead to increased arousal and activation of the SNS [61]. All these aspects can increase epinephrine and/or cortisol levels but also influence aldosterone, GH, and prolactin levels and other mediators such as prostaglandins or neurotransmitters driving process C (norepinephrine, dopamine, serotonin, and histamine) [45, 62, 63]. This symphony of mediators adds a further level of complexity to the neuroendocrine regulation of rhythmic immune-cell traffic during sleep and wakefulness. GαsPCR other than the β_2_AdR, such as DP_1_, EP_2/4_, IP, D_1/5_, 5-HT_4/6/7_, H_2_, CRF_1/2_, or MC_1-5_ receptors, likely show joint actions with cortisol and epinephrine on the anti-inflammatory cAMP/PKA pathway during daytime activity and low activation during nocturnal sleep [24, 25, 36, 63] and thus may also play an important role in this context.

Finally, leukocyte subsets with a cell-count rhythm peaking at night might be under the control of the parasympathetic nervous system [11], which is also influenced by sleep [64]. In addition to these neural and endocrine changes, sleep is accompanied by long-lasting recumbency and reduced locomotor activity, which suppresses the nocturnal nadir in core body temperature and impacts blood flow in the macro- and microvasculature [65]. Significant influences of temperature [36] and blood flow [21] on T-cell traffic are likely but have not been studied so far.

## Interplay of AdR and GR with leukocyte-intrinsic clock genes

Leukocyte-intrinsic clock genes may play a role in the neuroendocrine regulation of leukocyte traffic in humans by modulating AdR and GR expression, function, or downstream signaling events in immune cells. In vitro experiments with human cancer cell lines have shown that clock genes can regulate effects of GR and β_2_AdR [66, 67], and there is some evidence that clock genes also modulate GR effects in human leukocytes [68]. In human CD4^+^ T cells, rhythmic expression of clock genes, such as *PER3* and *REV-ERB α*, has been reported ex vivo and in vitro and was linked to rhythms in stimulated cytokine production [69]. The impact of T cell-intrinsic clock genes on AdR and GR, however, is currently unclear. The other directionality of interactions between receptors for stress mediators and clock genes is well known: Catecholamines and glucocorticoids can reset peripheral clocks, a function that is employed in the serum shock procedure to synchronize cells in culture [70]. Increases in *PER1* expression were shown in neutrophils [71] and peripheral blood mononuclear cells [72] after administration of glucocorticoids and in leukocytes after administration of β_2_AdR agonists [73]. An acute bout of exercise changed clock gene expression in CD4^+^ and CD8^+^ effector memory T cells [74], and clock genes, as well as genes involved in immune pathways, were acutely affected by one night of sleep deprivation [75] and a 10-h delay of the habitual sleep period [76]. Taken together, leukocyte-intrinsic clocks can be modulated by sleep and stress mediators. Such changes in leukocyte-intrinsic clocks may in turn play a downstream modulatory role in the neuroendocrine regulation of rhythmic leukocyte traffic, e.g., by regulating cellular processes upon activation of GR and AdR. However, further experiments are clearly needed to investigate the specific role of leukocyte-intrinsic clocks in modulating sleep-associated leukocyte rhythms in humans.

## Leukocyte numbers versus functions

We focused here on leukocyte counts as robust, validated blood parameters in medicine with clear 24-h rhythms, which have been known for decades. Availability of leukocytes determines host defense [77]; given that immune function is often related to frequency of immune cells, stress systems regulating the guided traffic of suitable immune cells to the site of action could also have a major impact on 24-h rhythms of functional markers of immunity [31, 78]. With respect to T-cell traffic, β_2_AdR and GR activation seems to converge on cAMP/PKA/Src signaling [33, 35], which is also known to suppress TCR activation and T-cell functions [79]. Moreover, β_2_AdR and GR activation inhibit the inflammatory NF_κ_B pathway [17, 67] and cytotoxicity [3, 63]. As cellular composition of a blood sample can change profoundly during the sleep–wake cycle (e.g., the ratio of CD8^+^ naive T cells to T_EMRA_ is 10:1 at 2 AM and 4:1 at 2 PM; Fig. 3 [27]), rhythms in functional readouts might reflect increases or decreases in counts of the subpopulation exerting this particular function. Therefore, functional markers should be assessed in characterized subpopulations, using e.g., isolation procedures or flow cytometry. Despite this limitation, most studies found that stimulated production of pro-inflammatory or Th1 cytokines and NK-cell cytotoxicity peak at night [e.g., 3, 80]. Thus, nocturnal sleep seems to support pro-inflammatory processes of recognition and response, whereas during daytime, homeostatic regulation, and resolution predominate, at least at a systemic level [46]. Locally, e.g., in tissues under attack, catecholamines may act in the opposite direction and support inflammation involving αAdR on innate immune cells [16, 17, 20, 81]. From an evolutionary point of view, it makes sense that local innate immune processes are boosted during the active phase (i.e., daytime in humans) as a first line of defense upon pathogen encounter, which is more likely to occur at this time of the 24-h cycle. On a systemic level, however, it might be important that anti-inflammatory processes occur in parallel to restrict potentially dangerous inflammatory processes in time and space. The short-term suppression of cytotoxic functions in effector cells mobilized into the circulation by catecholamines during a stress response might serve to prevent collateral damage induced by the mobilized killer cells on their way to the intended operation site. In contrast to the first line of defense, more sophisticated and slower processes, such as the formation of antigen-specific memory by T and B cells as the second line of defense, do not have to occur immediately upon pathogen encounter and therefore can be promoted during the inactive sleep phase. The circadian system may anticipate pathogen encounter, whereas sleep, and especially SWS, which increases following infections, may help to save energy for reallocation to the activated immune system. Together, the circadian system and sleep may synergize compatible and separate incompatible immunological processes to reduce costs and increase fitness [82].

## Other time dimensions and influences relevant for immune-cell rhythms

Immunological processes not only show 24-h rhythms but also weekly and annual fluctuations [3, 83]. Neutrophils peak in winter when lymphocyte counts are lowest [83]. Interestingly, integrin-driven adhesion and host defense functions of neutrophils show a maximum during summer [84], i.e., when neutrophil numbers in blood are low. These findings indicate that leukocyte numbers and functions are also adapted to environmental rhythms that are longer than 24 h. Yet, the role of sleep and stress mediators in this context still needs to be elucidated.

Ongoing stressors or long-lasting increases in stress mediators can exert effects on leukocyte traffic that completely differ from those of short-term stress and thus from physiological processes. Several lines of evidence indicate a reduced sensitivity of β_2_AdR or GR during chronic stress or even a resistance against the physiological anti-inflammatory actions of epinephrine or cortisol [85, 86]. These pathological, maladaptive processes during chronic stress can lead to systemic chronic inflammation (SCI) [87] with increased neutrophil and reduced lymphocyte counts [88] and impairments in pathogen and tumor defense [89].

Similar neuroendocrine-immune alterations become evident during aging [7]. Immune changes in the elderly are summarized as inflammaging or immunosenescence and encompass SCI, loss of Tearly due to thymic involution, inflation of senescent Tlate, and immunodeficiency [90, 91]. Notably, chronic stress and aging can be accompanied by impairments of sleep and the rhythmic regulation of the HPA axis [15, 92]. However, further experiments are necessary to investigate whether these findings reflect simple correlations or causes and consequences between sleep, stress systems, and immune functions.

## Findings from different animal species

Veterinarians are familiar with stress leukocytosis in pets, wildlife, and farm animals. The neutrophil-to-lymphocyte ratio can be used as a proxy for acute stress, because it increases in parallel with endogenous catecholamine and glucocorticoid levels [93], e.g., when capturing and transporting rhinoceroses [94]. Like humans, pigs show opposing rhythms in blood naive T-cell and NK-cell counts, in antiphase and in phase with cortisol, respectively [95]. Likewise, monkeys [96], rats [97], and guinea pigs [30] can be suitable laboratory models for the assessment of the effects of stress mediators on immune-cell numbers. Guinea pigs are rodents that share with humans a relative resistance to the apoptotic effects of glucocorticoids [30]. The situation is less clear in mice, as these animals are very sensitive to glucocorticoids and as, to our knowledge, the epinephrine-induced acute mobilization of murine stress leukocytes with increases in blood numbers was never demonstrated. Furthermore, in mice various blood leukocyte subsets have rhythms with peaks during the early rest period, even neutrophils and NK cells [98], which differs from findings in humans (Fig. 1). On the other hand, glucocorticoid-driven T-cell rhythms in murine blood have been known for decades [99]. Rhythmic bone marrow egress and re-entry were delineated in murine stem cell traffic, involving CXCR4, CXCL12, corticosterone, AdR activation, and sleep-dependent GH release [37, 100, 101]. However, experiments on rhythmic traffic of T cells in mice did not analyze the bone marrow compartment so far, although, like in humans [32], GR-induced increases in CXCR4 have later been demonstrated to drive circadian oscillations in T-cell distribution also in mice [102]. In humanized mice inverse patterns of clock gene-CXCR4 interaction were delineated for murine and human leukocytes [103], complicating the translation of mouse findings to humans. In guinea pigs, the assumed redistribution of T cells to the bone marrow upon GR activation was confirmed [30]. Also, glucocorticoid-CXCR4-driven traffic of eosinophils into the bone marrow was verified in monkeys, although it could not be demonstrated in mice [96].

In contrast to humans, mice are active during the dark period and sleep during the light period. Accordingly, corticosterone levels in blood peak at the transition from the rest to the active period in the evening [104] but are only seldomly assessed in analyses of immune rhythms. Certain mouse strains are deficient of melatonin and in those strains that are melatonin-proficient, the “hormone of darknessˮ peaks during the active period [104] and not during sleep as in humans [5]. Blood levels of murine catecholamines have to our knowledge not been measured along the sleep–wake cycle, making it overall difficult to assess the role of epinephrine for immune rhythms in mice with the current literature. Blood sampling is a stressful procedure and is in mice often done under general anesthesia, which itself can affect blood levels of stress mediators [105].

The investigation of the specific role of sleep in immune parameters in non-humans is even more difficult than the investigations of 24-h rhythms, because animals unlike humans do not refrain from sleep voluntarily. If kept awake by gentle handling—the gold standard for sleep deprivation without activation of the stress systems in mice—achieved wakefulness always goes along with explorative behavior, physical activity and, depending on the experimental setup, with food intake. The acute effects of sleep deprivation by gentle handling on counts of murine blood monocytes are strong [106] and clearly exceed the effects of circadian regulation of monocytes [98]. However, sleep compared to sleep deprivation increased classical Ly6C^hi^ monocytes in blood of mice [106], while in humans a decrease of total and non-classical CD16^+^ monocytes and no change in classical CD14^+^CD16^−^ monocytes has been observed [1, 12], again demonstrating diverging findings in human and murine data in neuro-immune interactions.

Despite these limitations, mouse models can certainly add to our understanding of neuro-immune interactions. For example, recent mouse studies showed clear anti-inflammatory effects of SNS activation on immune-cell traffic and function [e.g., 107]. However, selection of suitable mouse strains, adequate housing conditions (e.g., thermoneutrality, enriched environment, adequate group size), stress-minimizing blood sampling and killing procedures, short-term and thus more physiological manipulations of stress systems, and additional readouts including sleep, other behaviors (nesting, burrowing, locomotion, food intake), and blood parameters are important considerations when investigating circadian and neuroendocrine regulation of immune parameters in animal models [2, 108].

## Conclusions, clinical implications, and outlook

A rise in symptoms of inflammation, pain, and itch during or following the nocturnal sleep period is obvious for immune-driven diseases such as rheumatoid arthritis, bronchial asthma, or other allergic conditions [3, 70]. This is well in line with the presented research on 24-h rhythms in immune parameters indicating pro-inflammatory actions during nocturnal sleep. These clinical symptoms often interfere with sleep duration and quality. Resulting chronic sleep disturbances and stress could further disrupt the fine-tuned neuroendocrine regulation of the immune system along the sleep–wake cycle and lead to a vicious circle with resistance of β_2_AdR or GR, failures in anti-inflammatory control by stress mediators and further inflammation [46]. Clinical routines should therefore be adjusted to reduce current and further sleep impairments and circadian disruption of inpatients [109]. Wise timing of symptom-alleviating medication is used in first applications of chronopharmacology. For example, modified-release prednisone taken by patients with rheumatoid arthritis before bedtime exerts its anti-inflammatory actions in the mid-sleep, when it is needed most [110]. Given that lifestyle and psychosocial stressors are assumed to be key drivers of SCI and associated metabolic, cardiovascular, mental, neurodegenerative, and autoimmune diseases [87], several non-pharmacological interventions such as psychotherapy, physical exercise, and physical therapy may help to recalibrate β_2_AdR or GR and thus restore the immunoregulatory capacities of stress mediators. Finally, females and males differ in sleep patterns, immune parameters, and functions, and in negative health outcomes in response to chronic stressors, calling for studies assessing the effects of sex, gender, and menstrual cycle on immune rhythms [46, 92]. Overall, we hope that our review will stimulate future studies that simultaneously assess behavior in parallel with neuroendocrine and immunological blood parameters to delineate the rhythmic regulation of our immune system and its dysregulation in diseases.

## Methodological pitfalls



## Glossary

**The term 24-h rhythms** is used in our review globally when referring to rhythms with a period of 24 h (including endogenous circadian rhythms and rhythms without further specification of the origin). In most studies that we review here, 24-h rhythms were assessed during a regular sleep–wake cycle or during 24 h of continuous wakefulness. Free-running circadian rhythms in humans can be measured in sophisticated experimental protocols (e.g., isolation from external time cues or forced desynchrony, see box “Methodological pitfallsˮ). These were used to determine circadian rhythms of the sympathetic nervous system (SNS) and the hypothalamus–pituitary–adrenal (HPA) axis but to our knowledge not yet to study circadian regulation of leukocyte numbers.

**The terms day or daytime** are used in our review for the time period corresponding to the regular activity period (e.g., from 7 AM to 11 PM). Accordingly, a rhythm that peaks during this time period is referred to as daytime rhythm and color-coded in the figures in light blue. If the regular sleep–wake cycle was not experimentally manipulated, we also use the term daytime activity, indicating that effects might reflect circadian effects, effects of activity/wakefulness, or both.

**The terms night or nocturnal** are used in our review for the time period corresponding to the regular sleep period (e.g., with lights off from 11 PM to 7 AM). Accordingly, a rhythm that peaks during this time period is referred to as nocturnal rhythm and color-coded in the figures in dark blue. During a regular sleep–wake cycle, we also use the term nocturnal sleep, indicating that effects might reflect circadian effects, effects of sleep, or both (if not explicitly specified further). If a parameter was measured under conditions of experimental sleep deprivation, we use the term nocturnal wakefulness.

**The circadian system** is an internal timing system with an intrinsic period of around 24 h. It consists of cellular clocks in the brain and the periphery. The molecular basis of these clocks is constituted by so-called clock genes and clock-controlled genes, such as *PER1/2/3* and *REV-ERB α*, which have an expression-repression cycle of circa 24 h (for more details see other manuscripts of this issue). The hypothalamic master clock in suprachiasmatic nuclei (SCN) entrains peripheral clocks via, among others, neuroendocrine mediators, such as epinephrine and cortisol.

**Adrenoceptors** (AdR) are fast-acting transmembrane G-protein coupled receptors (GPCR) with different G protein α subunit types. The three βAdR (β_1_AdR, β_2_AdR, β_3_AdR) are GαsPCR, which activate the cyclic adenosine monophosphate (cAMP)/protein kinase A (PKA) signaling pathway. In contrast, α_2_AdR are Gαi/oPCR, which show opposite effects with reduced cAMP/PKA signaling. Finally, α_1_AdR are Gαq/11PCR, which enhance intracellular Ca^2+^. Effects of AdR activation on cell functions, e.g., on adhesion, occur within seconds to minutes.

**Glucocorticoid receptors (GR) and mineralocorticoid receptors (MR)** are cytoplasmic receptors that translocate into the nucleus to regulate gene expression and transcription into mRNA. Changes in protein translation then emerge with a certain delay. Apart from this genomic signaling, also faster atypical, e.g., non-genomic, signaling pathways were described [18, 31].

**Pro-migratory molecules** on immune cells are adhesion molecules such as integrins and selectins, and chemokine receptors, as well as their respective ligands, e.g., on endothelial cells. Selectins capture circulating leukocytes that are attracted to the endothelium by chemokines, leading to rolling of the leukocytes on the blood vessels. Chemokine receptor signaling on rolling leukocytes then induces integrin activation, leading to firm adhesion of the leukocytes to the vessel walls before they transmigrate into surrounding tissues. Re-entry of leukocytes into the circulation is among others driven by reversal of chemokine gradients or integrin de-activation [24, 77].

## Data Availability

Not applicable.
